# On the Hydrogen Bond Strength and Vibrational Spectroscopy of Liquid Water

**DOI:** 10.1038/s41598-018-35357-9

**Published:** 2018-11-15

**Authors:** Deepak Ojha, Kristof Karhan, Thomas D. Kühne

**Affiliations:** 1 Dynamics of Condensed Matter and Center for Sustainable Systems Design, Chair of Theoretical Chemistry, Paderborn University, Warburger Str. 100, D-33098 Paderborn, Germany; 2 Paderborn Center for Parallel Computing and Institute for Lightweight Design, Paderborn University, Warburger Str. 100, D-33098 Paderborn, Germany

**Keywords:** HB Strength, Time-frequency Correlation Function (FTCF), Stretch Frequency, Three-pulse Photon Echo, Frequency Fluctuations, Computational chemistry, Molecular dynamics

## Abstract

In the present work, we introduce two new metrics i.e. hydrogen-bond strength and charge-transfer between the donor/acceptor water molecules as a measure of hydrogen-bond rearrangement dynamics. Further, we also employ a simple model based on energy flux through the donor-acceptor water pairs to quantify the extent of the local hydrogen-bond network reorganization. Most importantly, we report a linear relationship between the OH stretch frequency and the charge and energy transfer through donor-acceptor water pairs. We demonstrate that the vibrational frequency fluctuations, which are used to determine third-order non-linear spectroscopic observables like the short-time slope of three pulse photon echo, can be used as an analog of the fluctuations in the hydrogen-bond strength and charge-transfer. The timescales obtained from our hydrogen-bond strength correlation and charge-transfer correlation decay are in excellent agreement with the computed frequency-time correlation function, as well as with recent vibrational echo experiments.

## Introduction

The role of liquid water in several physical, chemical, biological, atmospheric and geophysical processes has been extensively studied^1–3^. In liquid water, each water molecule forms multiple hydrogen-bonds (HBs) with its neighbors that leads to the formation of a strong HB network. The spatial and temporal evolution of the HB network is often responsible for many of the observed anomalous properties of liquid water. While the time-averaged local structure can be easily studied using X-ray and neutron scattering experiments^4–7^, the time-dependent evolution of the local solvent structure has been successfully elucidated with the advent of recent non-linear vibrational spectroscopic techniques like three-pulse photon echo peak shift (3PEPS) and two-dimensional infrared spectroscopy (2D-IR)^8–14^. To support these non-linear spectroscopic studies, several numerical and molecular dynamics (MD) studies have also been performed, which have helped to shed more information about the detailed mechanism of HB rearrangement^15–25^. In most of these studies, vibrational dephasing is associated with the memory loss of the electric field or the electrostatic force projected along on OH/OD modes.

Recently, the condensed-phase energy decomposition analysis based on absolutely localized molecular orbitals (ALMO-EDA) has been utilized to determine the strength of donor-acceptor interactions of individual HBs in liquid water^26–29^. Specifically, ALMO-EDA involves the separation of the total interaction energy into several physically meaningful components like donor-acceptor interactions, polarization of electron clouds between two molecules and some higher-order relaxation terms.

In the present work, we employ the ALMO-EDA method to investigate the relationship between the vibrational fluctuations of the OH stretching modes with the HB charge-transfer and corresponding energy stabilization in liquid water. While the HB strength and charge-transfer is computed using the ALMO-EDA technique, the instantaneous fluctuations in the ground-state frequencies of the OH modes are calculated using the Wavelet method of time-series analysis^21–23^. The calculation of nonlinear vibrational spectrum including non-Condon effects necessitates the determination of response functions that are expressed in terms of four-point dipole correlation functions^30^. Due to the fact that these calculations requires too much statistics to be viable within the employed ab-initio MD framework, the slope of three-pulse photon echo (S3PE), which is a theoretical analog of 3PEPS experiments, is calculated within the second-order cumulant approximation^31–33^. The ability of non-linear spectroscopic observables of the OH modes to capture fluctuations within the HB strength and charge-transfer is also investigated.

The frequency fluctuation dynamics of the OH modes in liquid water is often connected to the reorganization of the local solvent network. However, being a collective phenomenon, it is difficult to quantify the extent of reorganization in the local HB network. Therefore, we use a simple model based on the energetic balance to propose a novel linear relationship that permits to quantitatively predict the extent of reorganization in the HB network corresponding to a given stretching mode. In addition, the decay of the frequency-time correlation function (FTCF) is compared to the here proposed HB strength correlation function (HBCF), charge-transfer correlation function (CTCF) and local solvent reorganization index (LSRI). Furthermore, we compare these metrics with other more traditional approaches based on the HB lifetime (*S*_*HB*_(*t*))^34,35^, as well as HB number fluctuations (*N*_*HB*_(*t*))^36^.

## Results

### ALMO Energy Decomposition Analysis

In spite of the fact that the ALMO-EDA method has been described in detail elsewhere^26–29^, in the following we will briefly review its key concepts. In particular, the ALMO-EDA technique is a first-principles based approach to decompose the molecular binding energies into physically meaningful components. For that purpose, the total molecular binding energy (Δ*E*_*TOT*_) is segregated into the interaction energy of unrelaxed electron densities of the molecules (Δ*E*_*FRZ*_) and the density relaxation energy. The latter can be further decomposed into an intramolecular polarization contribution (Δ*E*_*POL*_) that is associated with the deformation of electron clouds on the molecules within their mutual fields, two-body donor-acceptor interactions (Δ*E*_*DEL*_) and a very small amount of higher-order relaxation terms (Δ*E*_*HO*_). The two-body donor-acceptor interactions arise due to the delocalization of electrons from the occupied orbitals of a donor molecule D to the virtual orbitals of an acceptor molecule A (Δ*E*_*D*→*A*_). Thus, the total energy can be written as the sum of the aforementioned terms as computed by the ALMO-EDA method, i.e.

1a $${\rm{\Delta }}{E}_{TOT}={\rm{\Delta }}{E}_{FRZ}+{\rm{\Delta }}{E}_{POL}+{\rm{\Delta }}{E}_{DEL}+{\rm{\Delta }}{E}_{HO},$$

where

1b $${\rm{\Delta }}{E}_{DEL}=\sum _{A,D=1}^{Mol}{\rm{\Delta }}{E}_{D\to A}.$$

The configurations for the ALMO-EDA analysis were obtained from our ab-initio MD trajectory. More precisely, 5000 equally distributed configurations separated by 5 fs each were extracted to compute the HBCF and CTCF correlation decay.

### Frequency Fluctuations Analysis

The instantaneous fluctuations of the ground-state frequencies of the intramolecular OH modes are computed by means of the Wavelet method of time-series analysis^19,21,22,37^. This method is based on the principle that a time-dependent function can be expressed in terms of basis functions obtained by the translations and dilations of a mother Wavelet

2 $${\psi }_{a,b}(t)={a}^{-\frac{1}{2}}\psi (\frac{t-b}{a}),$$

which is represented in the Morlet-Grossman form in the present study^38^. An important condition for the mother Wavelet to be applicable for time-series analysis is that it should rapidly decay to zero for *t* → ∞ and *t* → −∞. The coefficients of the Wavelet expansion are given by the Wavelet transform of *f*(*t*), i.e.

3 $${L}_{\psi }\,f(a,b)={a}^{-\frac{1}{2}}{\int }_{-\infty }^{+\infty }f(t)\bar{\psi }(\frac{t-b}{a})dt,$$

where *a* and *b* are both real quantities with *a* > 0. Here, *a* is the so called scale parameter and is directly related to the frequency content of the system over a small time interval around *t* = *b*. Therefore, depending upon the fluctuations in *f*, the wavelet transform *L*_*ψ*_*f*(*a*, *b*) gives the spectral information of the time-series *f* for the short time domain centered around *t* = *b*. Mathematically, for a wavelet *ψ* with centers t* and radius Δ_*ψ*_, *L*_*ψ*_ *f*(*a*, *b*) localizes the function within with the time window of domain [*b* + at* − *a*Δ_*ψ*_, *b* + at* + *a*Δ_*ψ*_]. Similarly, for a wavelet with its Fourier transform $\widehat{{\Psi }}$ and central frequency ω*, the frequency fluctuations are obtained within the domain of $[\frac{{\omega }^{\ast }}{a}-\frac{{{\rm{\Delta }}}_{\widehat{\psi }}}{a},\frac{{\omega }^{\ast }}{a}+\frac{{{\rm{\Delta }}}_{\widehat{\omega }}}{a}]$. Since the present wavelet method permits the simultaneous delocalization of frequency and time, it is employed to study non-periodic time-dependent fluctuations in the vibrational frequency of the OH stretching modes. The value of the scaling parameter *a*, which maximizes the modulus of the wavelet transform of *f* at time *t* = *b* is used to determine the most significant frequency component for the corresponding interval. The time-series, i.e. *f*(*t*), is constructed as a complex function with its real and imaginary parts corresponding to the instantaneous bond length and momentum of an OH mode projected along the OH bond. This method is then applied to all the OH modes present in a given system.

**Figure 1:**
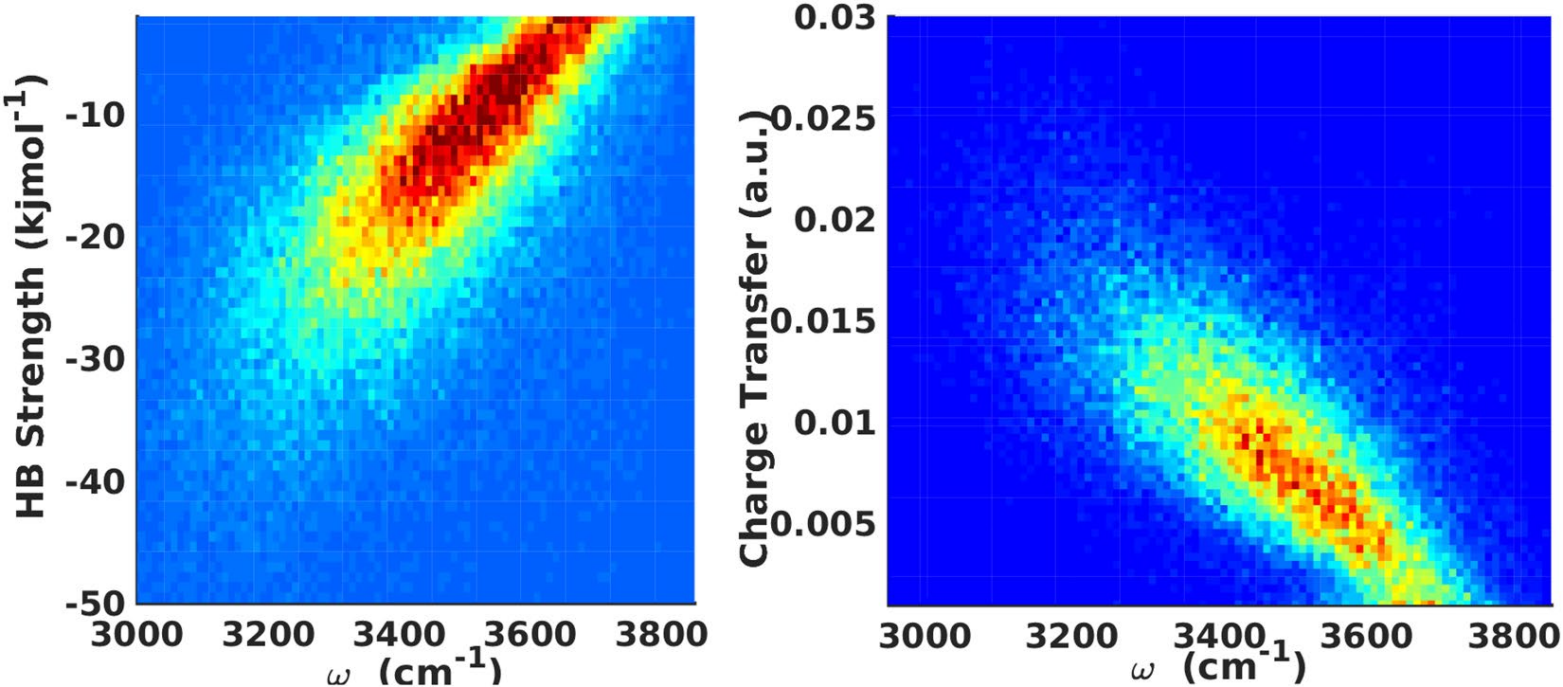
Time-averaged probability distributions of frequency versus (**a**) HB strength and (**b**) HB charge-transfer.

The probability distribution of vibrational frequencies of all OH stretching modes versus the energy Δ*E*_*D*→*A*_ and charge-transfer Δ*CT*_*D*→*A*_ from a donor molecule to an acceptor molecule through a HB are shown in Fig. 1. We note that on average, the vibrational frequency is inversely related to the HB strength (Δ*E*_*D*→*A*_) and HB charge-transfer (Δ*CT*_*D*→*A*_). Based on a simple least-square fitting, we obtain a linear relationship between the instantaneous vibrational OH stretch frequency and the corresponding HB strength and charge-transfer, i.e.

4a $${\rm{\Delta }}{E}_{D\to A}(kJ/mol)=0.0392-150.6\omega \,(c{m}^{-1})$$

4b $${\rm{\Delta }}C{T}_{D\to A}\,(a\mathrm{.}u\mathrm{.})=-\,0.0000221+0.086\omega \,(c{m}^{-1}),$$

where *ω* is the vibrational OH stretch frequency. The root mean square error (RMSE) for the two fits are 7.27 *kJ*/*mol* and 0.004 *a*.*u*., respectively. These linear relations can be easily used to determine the extent of charge-transfer and corresponding stabilization energy between a pair of hydrogen-bonded water molecules corresponding to a given vibrational OH frequency of an acceptor water molecule.

Several theoretical and experimental studies have shown that the loss of correlation within OH stretching modes is related to the HB rearrangement dynamics^14^. Nevertheless, it is not straightforward to quantify the extent of this local HB network reorganization due to its highly collective and spatially localized features. We therefore use a simple model based on the energetic balance to quantify the degree of solvent reorganization^39^. Specifically, for each H-bonded pair of water molecules, we compute the order parameter

5 $${O}_{h}={D}_{Acc}+{A}_{Don}-{D}_{Don}-{A}_{Acc},$$

where *D* denotes the energy transfer of the donor, whereas *A* identifies the acceptor water molecule. The subscript *Acc* signifies if the energy transfer is through the accepting and *Don* through the donating HB.

**Figure 2:**
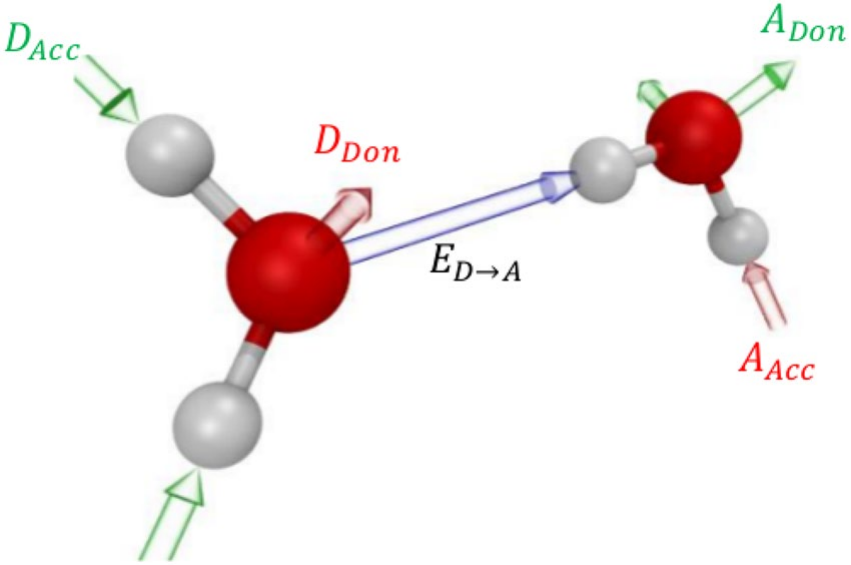
Illustration of different terms involved in the definition of the local solvent reorganization index in Eq. 5.

The nomenclature as described above is also illustrated in Fig. 2. However, since in the present work *O*_*h*_ is used to capture the HB reorganization kinetics, we will use the term local solvent reorganization index (LSRI) instead of *O*_*h*_ from now on. Our LSRI is based on the concept that the HB network reorganization around a water molecule can be associated with the charge-transfer induced net energy flux through its HB acceptors/donors. As a consequence, this allows us to partition the energy flux of each hydrogen-bonded water pair into its *D*_*Acc*_, *A*_*Don*_, *D*_*Don*_ and *A*_*Acc*_ contributions as shown in equation 5.

**Figure 3:**
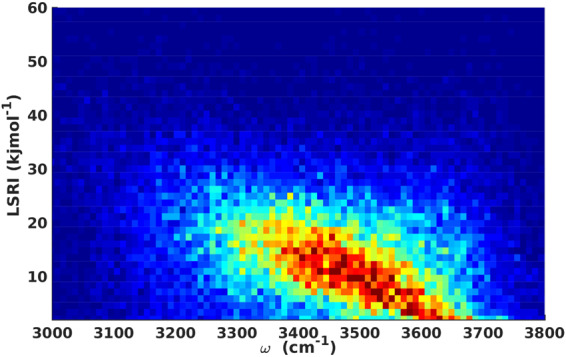
Time-averaged probability distribution of frequency versus LSRI.

With that, we are now able to determine the relationship between LSRI and the fluctuations in the vibrational frequency, as shown in Fig. 3. It is evident that the OH stretching frequency exhibits a strong dependence on the LSRI. Again, by means of a least-square fit of our computed data, we propose a very simple linear model that allows to predict the LSRI for a given frequency of the OH stretching mode, i.e.

6 $$LSRI\,(kJ/mol)=-\,0.01755+72.34\omega \,(c{m}^{-1}).$$

Previous MD simulations have shown a strong dependence of OH stretching frequency fluctuations on the local electric field/potential, as well as on the photon echo peak shift^15–18,30,32,33,36,40–43^. Similarly, experimental peak shift based studies have demonstrated that the time-evolution of the vibrational frequency can be used as a direct experimental probe of HB breaking/reformation^8,9^. Earlier, natural bond order analysis has also been used to provide an energy-based definition of hydrogen-bonding^44^. Our ALMO-EDA method, however, is also able to divide the total interaction energy into several chemical sensible components corresponding to many-body effects and donor-acceptor interactions^26–29^. The strength of this method is that it permits to rigorously quantify the amount of charge-transfer by locating the variationally lowest energy state without charge-transfer. Thereby, the issues of under/overestimation of charge transfer, contamination due to charge overlap effects are circumvented. Therefore, the time-dependent fluctuations in the energy Δ*E*_*D*→*A*_ and charge-transfer Δ*CT*_*D*→*A*_ can be used as a theoretical probe to observe the time-dependent evolution of hydrogen-bonding associated with a particular OH mode. In the next section, we demonstrate how these two-body interaction terms can be used to define new metrics to study the HB rearrangement dynamics.

## Discussion

Having demonstrated the existence of an overall correlation between the HB strength and charge-transfer and the LSRI with the OH stretching frequency, let us now examine the temporal decay of correlation in the LSRI and the HB strength and charge-transfer. The elegance of these metrics is again that they are solely based on the two-body delocalization-energy Δ*E*_*D*→*A*_, as well as charge-transfer Δ*CT*_*D*→*A*_ components, which can be readily used to study the HB rearrangement dynamics without incorporating any geometry-based criterion of a HB. For that purpose, we compute what we call the HBCF *C*_*HBs*_(*t*) and the CTCF *C*_*CT*_(*t*) that are defined as

7a $${C}_{HBs}(t)=\langle {\rm{\Delta }}{E}_{D\to A}(0)\cdot {\rm{\Delta }}{E}_{D\to A}(t)\rangle $$

and

7b $${C}_{CT}(t)=\langle {\rm{\Delta }}C{T}_{D\to A}(0)\cdot {\rm{\Delta }}C{T}_{D\to A}(t)\rangle ,$$

respectively.

**Figure 4:**
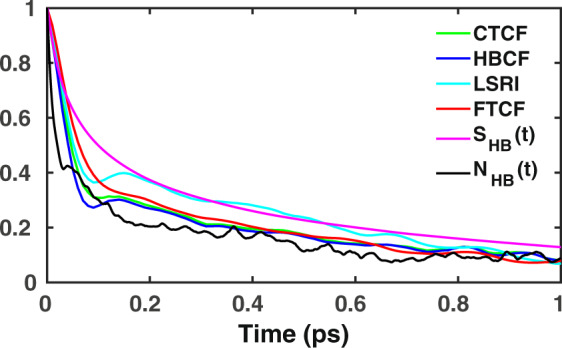
Time-dependent decay of fluctuations in the HB strength, HB charge-transfer, vibrational OH stretch frequency, continuous HB and HB number correlation functions, as well as the fluctuations in the LSRI.

The loss of correlation in the HBCF and CTCF exhibits a biphasic decay, as shown in Fig. 4. For both of the cases, the correlation decay shows a fast short-time decay within the initial 100 fs followed by a slower decay that extends up to a few picoseconds. For the purpose of extracting the timescales, the raw data of the HB strength and charge-transfer fluctuations are represented by a least-squares fit to the following bi-exponential function

8 $$f(t)={a}_{0}exp(-\frac{t}{{\tau }_{0}})+(1-{a}_{0})exp(-\frac{t}{{\tau }_{1}}),$$

where *τ*_0_ and *τ*_1_ are two characteristic time constants. In the case of the HBCF a strong oscillatory behavior is visible in short-term regime, which is less pronounced in the CTCF. In both cases, the decay patterns of both correlation functions are essentially overlappingly at long timescales, which is why the long-time decay constant *τ*_1_ was found to be 1.02 (±0.04) ps and 1.02 (±0.05) ps for both of them. The LSRI time-correlation function (LSRI-TCF) exhibits a slower initial decay, but a somewhat faster long-term decay with a timescale of 0.93 (±0.05) ps. Interestingly, *τ*_1_ of the HBCF, CTCF and LSRI-TCF are in very good agreement with the timescale of HB rearrangement, as reported in previous experimental and theoretical studies^10,23^. Interestingly, HBCF, CTCF and LSRI-TCF are not completely identical to each other. We note that our model, which is based on a linear fitting of HB strength, CT and LSRI as a function of vibrational frequency, entails a finite error bar for each of the metrics. Nevertheless, even considering serial correlation, the deviations observed in the time-dependent decay of these metrics are small enough to be within the provided error range.

In addition, we computed the FTCF for the OH stretch frequencies, which is also shown in Fig. 4. The long-term decay component was found to be 1.01 (±0.05) ps, which is in very good agreement with the timescales of our HBCF, CTCF and LSRI-TCF, respectively. The same also holds for the decay of the frequency fluctuations that is in agreement with the initial short-time and long-term decay of the HB strength and charge-transfer. As a consequence, it is evident that the vibrational frequency fluctuations and HB rearrangement can be used as a measure of the fluctuations in the HB strength due to HB charge-transfer between the donor and acceptor water molecules. The error bars were calculated using the block averaging method^45^. As the timescale of decay is nearly 1 ps, we divided the trajectory into three mini-trajectories of 8 ps length each in order to compute the timescale, as well as the corresponding error bar.

To test the applicability of our HBCF, CTCF and LSRI-TCF for aqueous solutions, we have compared them with more traditional approaches to quantify the HB dynamics such as continuous HB (*S*_*HB*_(*t*)) and HB number correlation (*N*_*HB*_(*t*)) functions^34–36^. The continuous HB correlation function determines the probability that an initially H-bonded pair of water molecules remains continuously intact until time *t*. The HB number correlation function is defined as

9 $$C(t)=\frac{\langle \delta n(t)\cdot \delta n(0)\rangle }{\langle \delta {n}^{2}\rangle },$$

where *δn*(*t*) = *n*(*t*) − 〈*n*〉 and *n*(*t*) is the number of HBs that a water molecule forms at time *t*. The time-dependent decay of *S*_*HB*_(*t*) and *N*_*HB*_(*t*) are also shown in Fig. 4. The timescales of correlation loss were again obtained using Eq. 8. The long-term decay component of *N*_*HB*_(*t*) was found to be 0.74 (±0.04) ps, which is substantially faster than that of FTCF. Similarly, the HB lifetime obtained from *S*_*HB*_(*t*) was found to be 1.2 (±0.04) ps. We find it important to note that in spite of the agreement between *S*_*HB*_(*t*) and *N*_*HB*_(*t*) with the FTCF, the former quantities can be utilized to determine the HB lifetime, but not to accurately predict the short-time decay dynamics.

Finally, we have examined the suitability of our HBCF and CTCF to determine spectral observables of non-linear vibrational spectroscopic techniques like S3PE, which is obtained from the integrated echo intensity

10 $$I({t}_{1},{t}_{2})={\int }_{0}^{\infty }d{t}_{3}{|\sum _{i\mathrm{=1}}^{3}{R}_{i}({t}_{1},{t}_{2},{t}_{3})|}^{2}.$$

The short-time slope of the integrated echo intensity at initial time *t* = 0 is defined as S3PE. More precisely,

11 $$S({t}_{2})=\frac{\partial I({t}_{1},{t}_{2})}{\partial {t}_{1}}{|}_{{t}_{1}=0}\mathrm{.}$$

**Figure 5:**
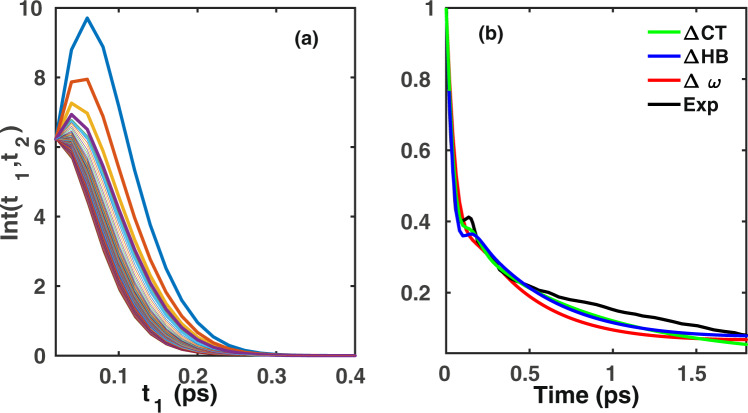
(**a**) Integrated vibrational echo intensity as obtained using the fluctuations in the HB strength and (**b**) S3PE obtained from the HB strength, HB charge-transfer, vibrational OH stretch frequency, as well as normalized 3PEPS obtained from ref.^43^.

The normalized S3PE, i.e.

12 $$C({t}_{2})=\frac{S({t}_{2})}{S\mathrm{(0)}},$$

is known to capture the loss of frequency correlation. The corresponding integrated echo intensity and time-dependent decay of S3PE, as obtained from the fluctuations of the HBCF, CTCF, FTCF and normalized 3PEPS deduced from mid-infrared spectroscopic experiments^43^, are shown in Fig. 5. We find that the S3PE as obtained from fluctuations within the HB strength and charge-transfer are much closer to the experimentally obtained 3PEPS than the S3PE computed from the frequency fluctuations. Interestingly, the initial short-time oscillatory trend that is seen in the experimental 3PEPS, although less pronounced in magnitude, is correctly reproduced in our calculations based on the HB strength fluctuations. As before, the corresponding timescales were obtained using the fit function of Eq. 8. The long-term decay constant of S3PE as obtained from the HB strength, charge-transfer and frequency fluctuations are 1.32 (±0.04) ps, 0.91 (±0.04) ps and 0.99 (±0.05) ps, respectively, and as such rather similar to the experimentally known value of the HB rearrangement dynamics. This clearly demonstrates that non-linear spectroscopic experiments can be used as a direct probe to determine the time-dependent evolution of the HB strength and charge-transfer.

## Summary and Conclusion

To summarize, in present work we have introduced the HB strength and HB charge-transfer between the donor/acceptor water molecules as a new metric of predicting the HB rearrangement dynamics. The great appeal of these metrics lies in the fact that they do not rely on any HB definition and can be readily obtained from ab-initio MD simulations. Based on that, we proposed a simple linear model to quantify the extent of HB reorganization using the ALMO-EDA energy flux through a donor-acceptor pair. We have also demonstrated the ablity of the introduced metrics to represent spectral observables of non-linear spectroscopic experiments like 3PEPS. The results obtained using the HB strength and HB charge-transfer are in better agreement with the experiments as compared to FTCF. Interestingly, HBCF also captures the short-time oscillatory pattern as reported in several infrared pump-probe experiments which is not seen in the decay of FTCF^43^. We conclude by noting that Eqs 4a,4b and 6 opens the door to experimentally quantify the donor-acceptor charge-transfer and the corresponding stabilization energy, as well as our LSRI by means of vibrational spectroscopy. Further, our model can also be employed to study the charge-transfer mediated HB stabilization in aqueous systems in general and at different surfaces, interfaces and also in confinement.

## Methods

The ab-initio MD simulation of liquid water was performed using the second-generation Car-Parrinello MD method^46–48^, as implemented in the mixed Gaussian and plane waves code CP2K/Quickstep^49,50^. Therein, the Kohn-Sham orbitals are expanded in contracted Gaussians, whereas the electronic charge density is represented using plane waves. For the former, we use an accurate molecularly optimized double-*ζ* basis set with one additional set of polarization functions (DZVP)^51^, while for the latter a density cutoff of 280 Ry was employed. The BLYP generalized gradient approximation to the unknown exchange and correlation functional was used together with Grimme’s D3 dispersion correction^52–54^. The core electrons, however, were represented by norm-conserving Goedecker-Teter-Hutter (GTH) pseudopotentials^55,56^. Previous works have shown that these settings provides a realistic description of many important structural, dynamical and spectroscopic characteristics of liquid water, including the partial pair correlation functions, self-diffusion and viscosity coefficients, HB lifetime, vibrational spectra and magnetic shielding tensor components^20,23,24,26,27,29^. The eventual system consisted of 64 light water molecules in a cubic box of length 12.43 Å corresponding to the density of liquid water at ambient conditions (300 K and 1 bar)^57^. The system was initially equilibrated in the canonical NVT ensemble for 10 ps followed by a production run in the microcanonical NVE ensemble for additional 25 ps.

## Data Availability

The data generated and analyzed during the current study are available from the corresponding author on reasonable request.
