# Correction: On the Hydrogen Bond Strength and Vibrational Spectroscopy of Liquid Water

**DOI:** 10.1038/s41598-026-61749-3

**Published:** 2026-07-30

**Authors:** Deepak Ojha, Kristof Karhan, Thomas D. Kühne

**Affiliations:** 1 Dynamics of Condensed Matter and Center for Sustainable Systems Design, Chair of Theoretical Chemistry, Paderborn University, Warburger Str. 100, D-33098 Paderborn, Germany; 2 Paderborn Center for Parallel Computing and Institute for Lightweight Design, Paderborn University, Warburger Str. 100, D-33098 Paderborn, Germany

Correction to: Scientific Reports 10.1038/s41598-018-35357-9, published online 15 November 2018

This Article contains errors in Equations (4a) and (4b), where the slope and intercept of the two linear fits are interchanged in the printed analytical expressions.

As a result, Equation (4a),

$$\Delta {E}_{D\to A}\left(kJ/mol\right)=0.0392-150.6\omega \left(c{m}^{-1}\right)$$

should read:

$$\Delta {E}_{D\to A}\left(\mathrm{k}\mathrm{J}{\mathrm{m}\mathrm{o}\mathrm{l}}^{-1}\right)=0.0392\omega \left({\mathrm{c}\mathrm{m}}^{-1}\right)-150.6$$

In addition, Equation (4b),

$$\Delta C{T}_{D\to A}\left(a.u.\right)=-0.0000221+0.086\omega \left(c{m}^{-1}\right),$$

should read:

$$\Delta C{T}_{D\to A}\left(\mathrm{a}.\mathrm{u}.\right)=0.086-0.0000221\omega \left({\mathrm{c}\mathrm{m}}^{-1}\right)$$

where, *(ω )* denotes the numerical value of the OH-stretch frequency in $({\mathrm{c}\mathrm{m}}^{-1})$. The corrected equations are consistent with Fig. 1a and Fig. 1b of the original Article: lower OH-stretch frequencies correspond to stronger hydrogen bonds, i.e. more negative $(\Delta {E}_{D\to A})$, and to larger charge transfer $(\Delta C{T}_{D\to A})$. The error was limited to the printed equations and does not affect Fig. 1, the underlying data, the analysis, or any conclusions of the Article.

Furthermore, the Acknowledgements section is incomplete, and it should read:

“The authors would like to thank the Gauss Center for Supercomputing (GCS) for providing computing time through the John von Neumann Institute for Computing (NIC) on the GCS share of the supercomputer JUQUEEN at the Jülich Supercomputing Centre (JSC). This project has received funding from the European Research Council (ERC) under the European Union’s Horizon 2020 research and innovation programme (grant agreement No 716142). The authors are deeply grateful to Prof. Dr. Poul B. Petersen for drawing their attention to this inconsistency. His recent work on vibrational spectroscopy of water and aqueous interfaces, in particular the Faraday Discussions article that cites and uses the hydrogen-bond-strength/OH-stretch-frequency relationship from the original Article, helped identify the mismatch between the plotted fits and the printed formulae.”

